# Rapid Production of Mn_3_O_4_/rGO as an Efficient Electrode Material for Supercapacitor by Flame Plasma

**DOI:** 10.3390/ma11060881

**Published:** 2018-05-24

**Authors:** Yang Zhou, Lei Guo, Wei Shi, Xuefeng Zou, Bin Xiang, Shaohua Xing

**Affiliations:** 1 School of Chemistry and Chemical Engineering, Chongqing University, Chongqing 400044, China; cquzhouyang@126.com (Y.Z.); njzouxf@126.com (X.Z.); 2 School of Material and Chemical Engineering, Tongren University, Tongren 554300, China; truweishi@163.com; 3 National-municipal Joint Engineering Laboratory for Chemical Process Intensification and Reaction, Chongqing 400044, China; 4 State Key Laboratory for Marine Corrosion and Protection, Luoyang Ship Material Research Institute (LSMRI), Qingdao 26623, China

**Keywords:** Mn_3_O_4_, reduced graphene oxide, supercapacitors, flame plasma

## Abstract

Benefiting from good ion accessibility and high electrical conductivity, graphene-based material as electrodes show promising electrochemical performance in energy storage systems. In this study, a novel strategy is devised to prepare binder-free Mn_3_O_4_-reduced graphene oxide (Mn_3_O_4_/rGO) electrodes. Well-dispersed and homogeneous Mn_3_O_4_ nanosheets are grown on graphene layers through a facile chemical co-precipitation process and subsequent flame procedure. This obtained Mn_3_O_4_/rGO nanostructures exhibit excellent gravimetric specific capacitance of 342.5 F g^−1^ at current density of 1 A g^−1^ and remarkable cycling stability of 85.47% capacitance retention under 10,000 extreme charge/discharge cycles at large current density. Furthermore, an asymmetric supercapacitor assembled using Mn_3_O_4_/rGO and activated graphene (AG) delivers a high energy density of 27.41 Wh kg^−1^ and a maximum power density of 8 kW kg^−1^. The material synthesis strategy presented in this study is facile, rapid and simple, which would give an insight into potential strategies for large-scale applications of metal oxide/graphene and hold tremendous promise for power storage applications.

## 1. Introduction

Supercapacitors have attracted much attention owing to their robust lifetimes, rapid charging capabilities and high power energy storage for mobile phone, hybrid electrical vehicles, back-up power systems, cranes, and lift [1,2,3,4,5]. A promising supercapacitor stores much more energy compared with electrostatic and electrolytic capacitors and exhibits stronger power with longer cycle life than the available battery [6,7].

Compared with a great mass of mercantile supercapacitor electrode, transition metal oxides used as supercapacitor electrode have high energy density, good capacitive performance and long cycle stability [8,9]. Manganese oxides represent a suite of transition metal oxides interesting scientific workers in energy storage field, because of their low cost, natural abundance, environmental compatibility and excellent pseudocapacitive property (~1370 F g^−1^) [10,11]. However, its low electrical conductivity which is about 10^−7^~10^−8^ S cm^−1^ remains one of the major challenges to be addressed. This drawback can be overcome by using hybrid materials via coupling of conductive materials and MnO_x_ [12,13]. Combining well-aligned nanocrystalline structures and two-dimensional (2D) graphene sheet with enhanced electron transport, ultrahigh exposed surface area and improved mechanical stability are extremely interesting [14,15,16,17,18].

Recently, MnO_*x*_/rGO composites as advanced electrode materials in ECs have been reported [19,20,21]. Wang et al. synthesized Mn_3_O_4_/reduced graphene oxide nanohybride paper using hydrothermal method. The obtained Mn_3_O_4_/rGO composite has kept promising cycle 95% of retention ratio after 6000 cycles. The composite delivers a volumetric energy density of 0.0055 Wh cm^−3^ and a high volumetric capacitance of 54.6 F cm^−3^ in a half-cell [22]. Wang and co-workers reported an ASC was assembled by graphene@MnO_2_ nanosheet hybrid materials as the positive electrode and porous graphene as the negative electrode, respectively. Graphene@MnO_2_ was prepared by polyaniline-assisted anchoring MnO_2_ ultrathin nanosheet and uniformly on the graphene substrate. The composite was used as an electrode show excellent specific capacitance of 245.0 F g^−1^ at 0.5 A g^−1^, as well as 74.5% of retention at large current density. Furthermore, the assembled ASC achieved a promising energy density of 7.9 Wh kg^−1^ at exceptional power density of 11,804 W kg^−1^ [23]. Liu et al. demonstrated a facile one-pot solvothermal process. Octylamine as surfactant, graphene sheets as substrate and manganese carbonyl (Mn_2_(CO)_10_) as precursor are prepared Mn_3_O_4_ nanodot composites anchored on nitrogen-doped graphene sheet (Mn_3_O_4_ NDs@NG). An ASC is based on the Mn_3_O_4_ NDs@NG and the assembled device exhibits high energy density of 90.7 W h kg^−1^ [24]. The results of these studies demonstrated the beneficial role of rGO and the significance of synergistic effects. Graphene is found as the thinnest two-dimensional and its basic structure unit is benzene six-membered ring. It is just a one carbon atom thick and honeycomb-like sheet showing lots of particular physical chemistry properties. Besides, heterogeneous nucleation and graphene used as templates to direct material deposition have been proven an effective way to guide the growth of novel nanomaterials [25,26]. In addition, MnO_*x*_/rGO composites could be assembled to be a free-standing electrode preventing the decrease in electrical conductivity caused by binders and keeping system stable. Various approaches have been proposed to synthesis MnO_*x*_/rGO hybrid materials, such as hydrothermal [27], chemical vapor deposition [10] and electrochemical deposition [28]. However, these methods show limitation in large scale preparation for high quality materials. There is great room for improvement in MnO_*x*_/rGO synthesis.

In this study, we report a simple, efficient, convenient, and facile technology for preparing the Mn_3_O_4_/rGO composite for promising performance supercapacitor electrode by using a naked flame of ethanol fuels. The optimized Mn_3_O_4_/rGO composite exhibits excellent specific capacitance of 342.5 F g^−1^ at current density of 1 A g^−1^ and exceptional cycle ability, demonstrating excellent electrochemical characteristics. Furthermore, an asymmetric supercapacitor (ASC) is designed using Mn_3_O_4_/rGO as the positive electrode, activated graphene (AG) as the negative electrode, and 0.1 M Na_2_SO_4_ as the electrolyte. The results show it can achieve a high cell voltage of 1.6 V and deliver a maximum energy density 27.41 Wh kg^−1^ and a power density of 8 kW kg^−1^. Such fabrication technique demonstrates features of simplicity, rapidity and low-cost, thus a hopeful approach for producing metal oxide/graphene, holding tremendous promise for power storage systems.

## 2. Experimental Section

### 2.1. Materials and Chemical

Graphite powder (99.99%) were purchased from Qingdao Jinrilai graphene corporation (Qingdao, China). Sodium nitrate (NaNO_3_, ≥98.0%), ethanol (CH_3_CH_2_OH, ≥98.0%), and aqueous hydrogen peroxide solution (H_2_O_2_, 30.0%) were obtained from Chengdu Kelong reagent factory (Chengdu, China). Potassium permanganate (KMnO_4_, 99.0%) and sulphuric acid (H_2_SO_4_, 98.0%) were purchased from Chongqing Chuandong chemical (group) Co., Ltd. (Chongqing, China) Manganese(II) acetate tetrahydrate (C_4_H_14_MnO_8_, >99.0%) was purchased from Sigma-Aldrich, Shanghai, China.

### 2.2. Preparation of the Mn_3_O_4_/rGO Composite

#### 2.2.1. Synthesis of Graphene Oxide (GO)

GO was synthesized by a modified Hummers method [29]. The preparation details of GO can be seen in the electronic supplementary information (Supplementary Materials S1).

#### 2.2.2. Synthesis of MnO_2_/GO Film

In detail, 400 mg of GO was dispersed in 100 mL of DI water by sonication for 1 h and transferred into three-necked 500 mL round-bottomed flask. 100 mL KMnO_4_ (0.05 M), followed by 100 mL Mn(Ac)_2_ solution (0.05 M) was carefully added into the GO suspension. The reaction proceeded under a controlled temperature of 90 °C for several hours to ensure full reaction, and finally cooled to the room temperature. Then, the samples were collected and washed several times with deionized water. They were dried at 35 °C under vacuum and MnO_2_/GO film was obtained.

#### 2.2.3. Synthesis of Mn_3_O_4_/rGO Film

The MnO_2_/GO hybrid film quickly passed through a naked flame from a burning alcohol lamp (video, see the Supplementary Materials). The color of MnO_2_/GO film changed from yellow-brown to reddish brown, and Mn_3_O_4_/rGO film was obtained.

**Scheme 1:**
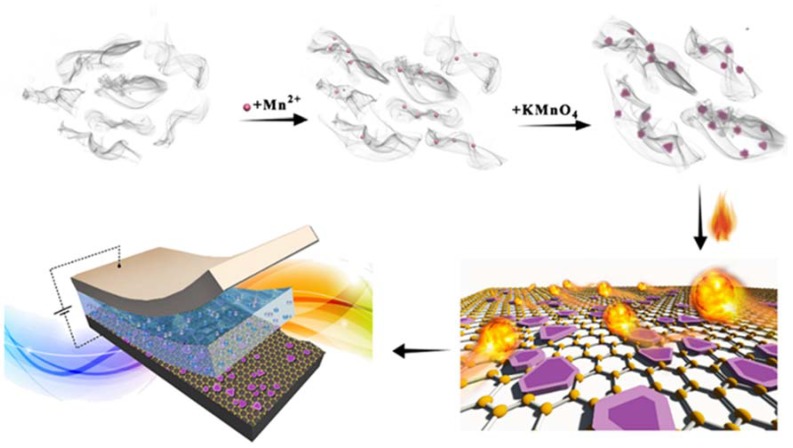
Schematic illustration of the synthesis process of the Mn_3_O_4_/rGO composite.

The overall synthetic route of Mn_3_O_4_/rGO is illustrated in Scheme 1.

### 2.3. Electrode Preparation

The obtained Mn_3_O_4_/rGO samples were ground into fine powders to press between two nickel foams (1 × 1 cm^2^) forming an electrode as the working electrode or positive electrode. In two-electrode system, the activated graphene (AG) was also pressed between two nickel foams to form the negative electrode. The preparation details of AG can be seen in the Supplementary Materials S2 section.

### 2.4. Materials Characterization

The sample IR spectra were studied using Nicolet iS50 FTIR (Fourier-transform infrared spectroscopy) spectrophotometer. Raman measurements were utilized with Raman spectrometer (LabRAM HR Evolution, Horiba, Kyoto, Japan). The phase composition was analyzed by an X-ray diffractometer (XRD, SuperNova, Agilent, Santa Clara, CA, USA). The chemical compositions were conducted on X-ray photoelectron spectroscopy (L Escalab 250Xi, Thermo Fisher Scientific, Hampton, NH, USA). Field emission scanning electron microscopy (FE-SEM) images were obtained on a JEOL JSM-7800F microscope (JEOL, Tokyo, Japan) and Transmission electron microscope (TEM) images were taken on Tecnai G2 20 microscopes (Fisher Scientific, Hampton, NH, USA).

### 2.5. Electrochemical Measurements

The electrochemical experiments were done in a classical three-electrode system which consists of working electrode, counter electrode, and reference electrode (SCE). All the electrochemical measurements were carried out with a CHI760E electrochemical workstation and repeated more than three times. The cyclic voltammetry (CV) analysis at various scan rates as well as the galvanostatic charge-discharge (GCD) at different current densities was performed in 0~0.8 V. Electrochemical impedance spectroscopy (EIS) was obtained by applying an AC voltage of 5 mV amplitude in the frequency range of 0.01~100 kHz.

## 3. Results and Discussion

**Figure 1:**
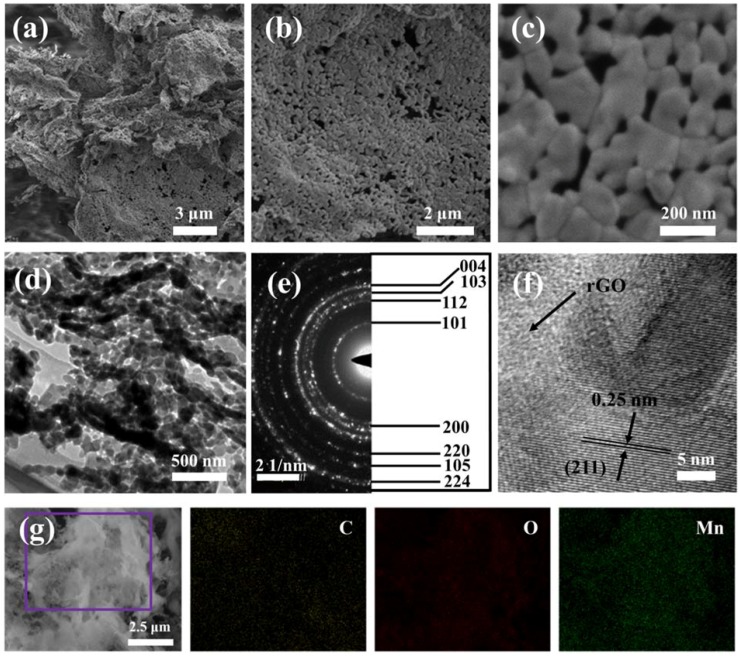
(**a**–**c**) FE-SEM; (**d**) TEM; and (**e**) is the SAED pattern of Mn_3_O_4_ with (211) in the Mn_3_O_4_/rGO, indicative of the well-textured and single-crystalline nature of Mn_3_O_4_ NPs; (**f**) HR-TEM images of Mn_3_O_4_/Rgo; (**g**) is the elemental mapping analysis of Mn_3_O_4_/rGO composites.

The SEM and TEM images of Mn_3_O_4_/rGO are shown in Figure 1. As can be seen from Figure 1a, the image acquired at the surfaces of Mn_3_O_4_/rGO shows Mn_3_O_4_ nanosheet evenly and fully anchored on the rGO layers. A high magnification SEM image (Figure 1b) exhibits that the Mn_3_O_4_ nanosheet with an average size of about 100 nm (Figure 1c) forms dense nanosheet area on graphene layers. The TEM image (Figure 1d) shows an overview of the Mn_3_O_4_/rGO samples. This image demonstrates that the lightweight conductive rGO with abundant wrinkles serves as an excellent conductive bracket for Mn_3_O_4_ nanosheet attachment. It is notable that, after a long time of high-power sonication before tested with TEM, the Mn_3_O_4_ nanosheet was still stably anchored on the graphene layer surface with high density, suggesting a strong interaction between graphene layers and Mn_3_O_4_ nanosheet. In addition, the nanosheet could act as a barrier to prevent the adjacent restack of graphene sheets, thus avoiding losing their high active surface area. The SAED pattern shows crystalline nature of Mn_3_O_4_ nanoparticles and the diffraction rings which could be attributable to the (004), (103), (112), (101), (200), (220), (105) and (224) planes of Mn_3_O_4_ nanosheet (Figure 1e). The planes are in accordance with the XRD results in previous reports [30]. The HR-TEM (Figure 1f) focuses on lattice planes of Mn_3_O_4_ nanosheet. The result of the interplanar spacing of the crystalline grains was 0.25 nm, closely matching with the (220) plane orientation [31]. Figure 1g shows the elemental mapping analysis of Mn_3_O_4_/rGO composites including C, O, Mn.

**Figure 2:**
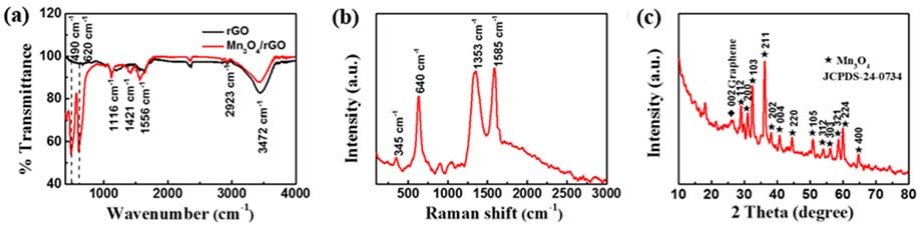
(**a**) FTIR spectra of rGO and Mn_3_O_4_/rGO; (**b**) Raman spectrum and (**c**) Powder XRD pattern of Mn_3_O_4_/rGO.

The FT-IR spectra of the rGO and Mn_3_O_4_/rGO in Figure 2a reveals that the stretching peak appears at 2923 cm^−1^ and 1421 cm^−1^ are attributable to C–H and C–C band, which refer to skeletal vibrations of non-oxidized graphitic domains [32]. The presense of peak at 1556 cm^−1^ belong to carbonyl group band could be attributed to the unreduced carboxy group. The peak at 1116 cm^−1^ suggested C–O vibrations of carboxylic acid. In addition, the new peaks (at 490 cm^−1^ and 620 cm^−1^) were observed, which was due to Mn–O stretching mode in tetrahedral site and octahedral environment. In addition, the absorption peak at 3472 cm^−1^ is ascribed to the O–H vibrations from adsorbed moisture.

Moreover, Mn_3_O_4_/rGO composite could be clearly verified by Raman spectra. Figure 2b shows that two distinct peaks occur at 1353 cm^−1^ (D-band: vibrations for dangling bonded carbon atoms) and 1585 cm^−1^ (G-band: vibrations for all sp^2^ bonded carbon atoms). Additionally, an obvious peak at 640 cm^−1^ and a small peak between 200 cm^−1^ and 400 cm^−1^ are in agreement with the data of Mn_3_O_4_ compound in previous reports [33]. Complementary XRD patterns of the Mn_3_O_4_/rGO provide an identification in chemical composition and crystalline structure (Figure 2c). A minor broad (002) diffraction peak appearing between 24.5° and 27.5° could be due to the disorderedly stacked graphene sheets. The diffraction peaks at 28.88°, 31.02°, 32.32°, 36.09°, 36.45°, 37.98°, 44.44°, 50.71°, 53.86°, 56.00°, 58.51°, 59.84° and 64.65° could be assigned to the (112), (200), (103), (211), (202), (004), (220), (105), (312), (303), (321), (224) and (400) planes, respectively, which unambiguously demonstrates the highly crystalline structure of Mn_3_O_4_ nanosheet (JCPDS Card No. 24-0734). With these spectral methods, chemical composition and the structure of Mn_3_O_4_/rGO could be identified.

**Figure 3:**
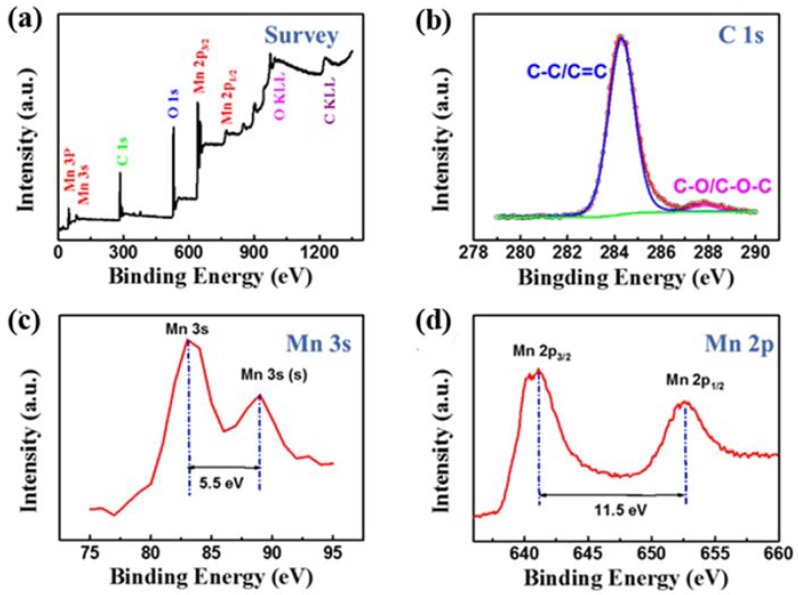
(**a**) XPS spectrum of Mn_3_O_4_/rGO, (**b**) C 1s, (**c**) Mn 3s and (**d**) Mn 2p XPS spectra of Mn_3_O_4_/rGO composite.

Further valence state information about Mn_3_O_4_/rGO hybrid was obtained from XPS characterization. The full XPS survey exhibits the presence of C, O and Mn (Figure 3a). The C1s peak was best fitted in with two sharp peaks centred on at 286.5 eV and 284.7 eV, which were attributable to binding energy of C–O/C–O–C and C–C/C=C bonds (Figure 3b) [34]. Additionally, the multiplet split of Mn 3s peak in Figure 3c characterized the manganese oxidation state. This split is attributed to the exchange interaction between remaining electrons in the 3s orbital and other unpaired electrons which have parallel spins. The splitting width (5.5 eV) is in consistent with Mn_3_O_4_ [35]. In the high resolution Mn 2p region, two peaks around at 641.1 eV and 652.6 eV were observed and their splitting width is 11.5 eV, which were ascribed to Mn 2p_3/2_ and Mn 2p_1/2_ (Figure 3d). The XPS results further confirm the Mn_3_O_4_ nanosheet was grown on the graphene layer [36].

**Figure 4:**
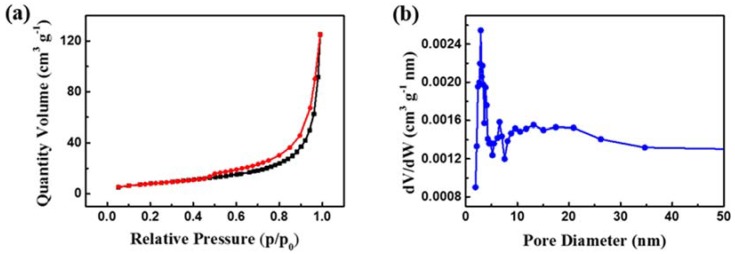
(**a**) N_2_ adsorption-desorption isotherms of Mn_3_O_4_/rGO and (**b**) the corresponding pore size distribution.

The porous nature of the Mn_3_O_4_/rGO was analysed by nitrogen adsorption-desorption isotherms (Figure 4a). The Brunauer Emmett Teller (BET) specific surface area of Mn_3_O_4_/rGO was 36.197 m^2^ g^−1^. In addition, the pore size distribution calculated from the Barrett-Joyner-Halenda method were concentrated at 3 and 5~15 nm (Figure 4b). The porous texture of Mn_3_O_4_/rGO material could be a benefit for the excellent electron and ion percolation network.

**Figure 5:**
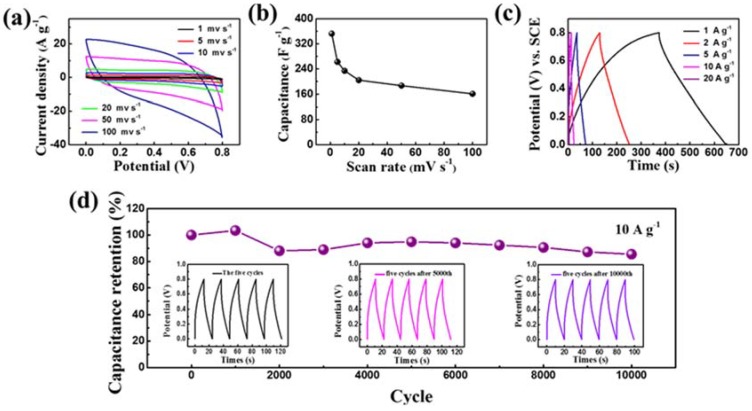
Electrochemical measurements in 0.1 M Na_2_SO_4_ solution. (**a**) CV curves of Mn_3_O_4_/rGO at various scan rates; (**b**) specific capacitance of the as-prepared Mn_3_O_4_/rGO material at various scan rates; (**c**) galvanostatic charge–discharge curves of Mn_3_O_4_/rGO at various current densities; (**d**) cycle performance measured over 10,000 cycles at a 10 A g^−1^.

Electrochemical characterization of the Mn_3_O_4_/rGO nanosheets was done by galvanostatic charging-discharging (GCD) and cyclic voltammetry (CV) at the room temperature of 25 °C. CV measurements of Mn_3_O_4_/rGO composite recorded at different scan rates were performed. The CV obtained from different scan rates are near-perfect rectangular without distinct redox peaks. This feature reveals that the effective capacitance is attributed to pseudocapacitance of the Mn_3_O_4_ nanosheet and double layer capacitance of rGO [34]. However, the CV curves gradually turn into a sharp tip towards 0.8 V with scan rate decreasing, because the charge mobility in porous structure is inhibited by the internal resistance of the electrode material. The CV shapes change little with scan rate increasing, suggesting good high-rate capability of the composite. The reason might be that graphene layer structure and the mesoporous structure of Mn_3_O_4_ nanosheet provide excellent electron conductivity and high channels for ion diffusion. The specific capacitance of Mn_3_O_4_/rGO calculated based on the CV curves are shown in Figure 5b (see Supplementary Materials S3 and S4 for calculation details).

A complementary measurement of the Mn_3_O_4_/rGO nanocomposite electrode capacitance was done by GCD studies over a voltage varying from 0 to 0.8 V. Figure 5c shows the GCD profiles of Mn_3_O_4_/rGO electrode samples at the different current densities. When the Mn_3_O_4_/rGO electrode was measured at 1, 2, 5, 10 and 20 A g^−1^, the specific capacitance could reach 342.5, 304.5, 223.1, 146.2 and 62.5 F g^−1^, respectively. (Calculation details see Supplementary Materials S5 and S6). The specific capacitance decreases with current density increasing. The result is in accordance with the preceding CV studies and illustrates excellent electrochemical performance of obtained Mn_3_O_4_/rGO material. Additionally, the Mn_3_O_4_/rGO electrode was subjected to 10,000 cycles of full-depth charge/discharge cycles at the large current density of 10 A g^−1^ with excellent retention of 94.87% over the whole 6000 cycles and even after 10,000 cycles it still keeps 85.47% of its initial capacitance (Figure 5d).

**Figure 6:**
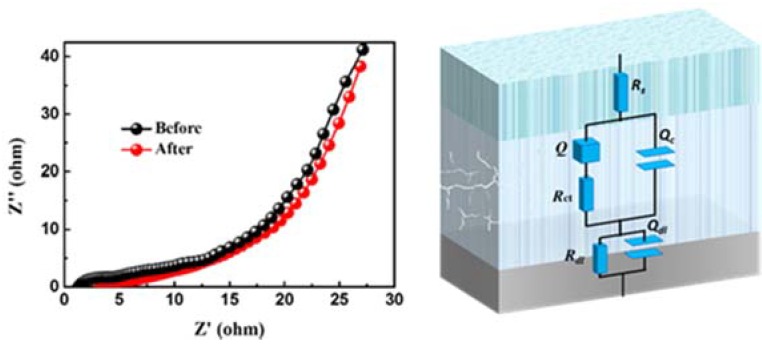
EIS spectra of Mn_3_O_4_/rGO measured at open circuit potential with frequency ranging from 0.01 Hz to 100 kHz in 0.1 M Na_2_SO_4_.

The impedances of Mn_3_O_4_/rGO before and after 10,000 whole cycles were also tested (Figure 6). *R*_s_ represents the equivalent series resistance (ESR) [37] consisting of intrinsic resistance, electrolyte resistance and contact resistance between current collector and Mn_3_O_4_/rGO. Here, the value of *R*_s_ changed slightly from 1.945 Ω to 2.544 Ω after 10,000 cycles, suggesting low internal resistance of the electrode and good conductivity of the electrolyte. In addition, just a slight increase of *R*_ct_ changed from 0.1776 Ω to 0.2085 Ω after 10,000 cycles. These results demonstrate that there is no apparent difference before and after 10,000 cycles, indicating ideal capacitive behavior.

**Figure 7:**
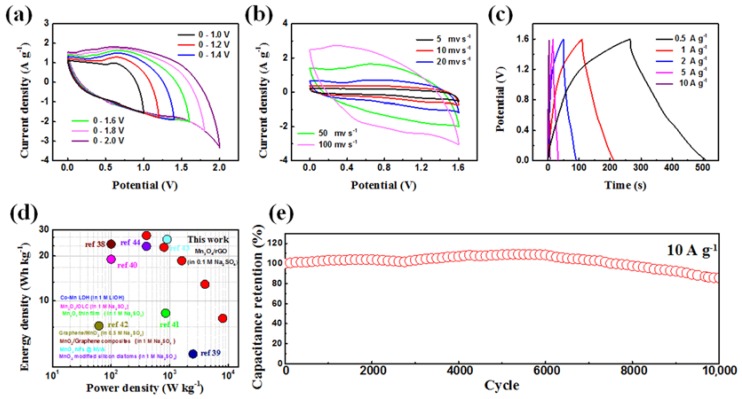
Electrochemical performance of an ASC in 0.1 M Na_2_SO_4_ solution. (**a**) CV curves at the scan rate of 50 mV s^−1^ with different potential windows; (**b**) CV curves measured at different scan rates with the potential window of 1.6 V; (**c**) GCD curves at different current densities with the potential window of 1.6 V; (**d**) Ragone plots of the ASC compared with other work; (**e**) cycle performance measured over 10,000 cycles at 10 A g^−1^.

To evaluate the Mn_3_O_4_/rGO composite electrochemical characteristics in practical application, an asymmetric supercapacitor device (ASC) device was assembled. The results of CV studies (1.0~2.0 V) are shown in Figure 7a and there is no obvious redox peaks and polarization with the voltage up to 2.0 V. The CV curves at potential window between 0 and 1.6 V show the same shape CV curves at different scan rates (Figure 7b), revealing excellent supercapacitor behavior and an unexceptionable fast charge-discharge property for ASC. According to mass loadings of the positive and negative electrodes, the device delivers a relatively high capacitance of 77.1 F g^−1^ (Figure 7c). As shown in Figure 7d, on basis of GCD curve at 0.5 A g^−1^, a high energy density (*E*) of 27.41 Wh kg^−1^ with a corresponding power density (*P*) value of 400 W kg^−1^ could be obtained. The maximal *P* could maintain 8 kW kg^−1^ with the *E* value maintaining as high as 7.8 Wh kg^−1^, which are larger than that of reported manganese oxide-based materials in the literature. The Mn_3_O_4_/rGO shows superior properties in terms of *E* and *P* over MnO_2_/rGO (24 Wh kg^−1^ at 100 W kg^−1^) [38], cobalt-manganese layered double hydroxide (4.4 Wh kg^−1^ at 2500 W kg^−1^) [39], Mn_3_O_4_/onion-like carbon (OLC) nanohybrid (19 Wh kg^−1^ at 100 W kg^−1^) [40], spray deposited hausmannite Mn_3_O_4_ thin film (8.27 Wh kg^−1^ at 850 W kg^−1^) [41], graphene/MnO_2_ (6.8 Wh kg^−1^ at 62 W kg^−1^) [42], MnO_2_ NFs@PPy NWs (25.8 Wh kg^−1^ at 901.7 W kg^−1^) [43], and MnO_2_ modified silicon (23.2 Wh kg^−1^ at 400 W kg^−1^) [44].

Long cycling ability is also a key factor in evaluating supercapacitor electrode material in practical applications. The GCD were tested with large current density of 10 A g^−1^ in a voltage window of 0~1.6 V and the results are shown in Figure 7e. Due to a gradual activation process of material, the specific capacitance of the Mn_3_O_4_/rGO electrode gradually increased during the first 6000 cycles, which was similar to the previous report [24,45]. The promising capacitance retention of 85.7% sustained after 10,000 cycles. Therefore, the above results demonstrated that the Mn_3_O_4_/rGO would be a kind of promising material for power supply devices.

**Figure 8:**
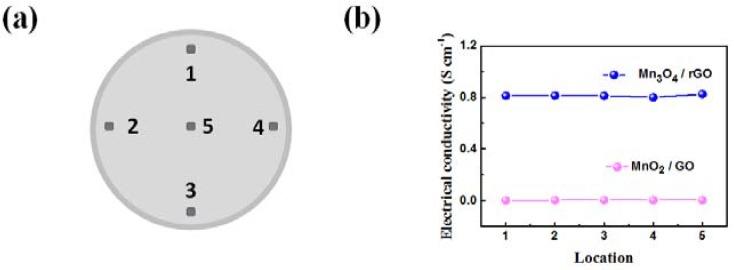
(**a**) Selected locations of MnO_2_/GO and Mn_3_O_4_/rGO film; (**b**) the electrical conductivity values of MnO_2_/GO and Mn_3_O_4_/rGO tested by 4-point probes resistivity measurement system, respectively.

Moreover, Mn_3_O_4_/rGO film was obtained from MnO_2_/GO through the flame reaction. The conductivity of MnO_2_/GO film and Mn_3_O_4_/rGO film was tested by a 4-point probes resistivity measurement system. The points on the surface of samples selected for testing conductivity were exhibited in Figure 8a. Obviously, as shown in Figure 8b, based on the conductivity value of the five points, the average electrical conductivity of MnO_2_/GO film and Mn_3_O_4_/rGO film were calculated to be 3.01 × 10^−3^ S cm^−1^ and 0.813 S cm^−1^, respectively, suggesting that the electrical conductivity of the MnO_2_/GO increased significantly through the flame reaction.

On basis of solution-based redox method, the MnO_2_ nanosheets were prepared and GO was employed as a template. The flame, as a special plasma, produces a gaseous mixture of H_2_ and CO through partial oxidation of the fuel with O_2_ from the ambient air, but also provides heat for a thermally sustained fuel cell (500–800 °C). The naked flame could enhance the process of decomposition, in which insulating functional groups over GO basal planes turned into CO_2_ and H_2_O. Meanwhile, the MnO_2_ nanosheets collapsed and turned into Mn_3_O_4_ which are evenly anchored on the surface of graphene sheet. This low-cost method simplifies the electrode preparation process. During the faradaic reaction, the binder-free electrode preparation method lowers the electrical resistance from binder and reduces mass/volume.

## 4. Conclusions

A well-dispersed Mn_3_O_4_ nanosheet anchored on reduced graphene (Mn_3_O_4_/rGO) composite was designed by a facile chemical co-precipitation and subsequent flame procedure. The obtained Mn_3_O_4_/rGO composite electrodes display fine pseudocapacitive performance.

(1) The Mn_3_O_4_/rGO electrode in the three-electrode system delivered high capacitance up to 342.5 F g^−1^ at the current density of 1 A g^−1^ and exhibited equally remarkable cyclic stability.
(2) The ASC consisting of an AG negative and a Mn_3_O_4_/rGO positive achieved a relatively high energy density 27.41 Wh kg^−1^ and a power density of 8 kW kg^−1^.
(3) Mn_3_O_4_/rGO material demonstrated in this paper can offer conductive porous texture for the excellent electron and ion percolation network.
(4) Such fabrication technique is simple, rapid, facile and low-cost and it would give an insight into producing the similar metal oxide/graphene as potential active materials for energy storage device.

## Supplementary Materials

The following are available online at http://www.mdpi.com/1996-1944/11/6/881/s1, Figure S1: The changes of the samples before and after the reaction triggered by the flame, Figure S2: Raman spectrums of MnO_2_/GO and Mn_3_O_4_/rGO, Figure S3: CV curves of Mn_3_O_4_/rGO and AG at 20 mV s^−1^, Figure S4: SEM (a,b) and Mn 2p XPS spectra of Mn_3_O_4_/rGO composite (c,d) of Mn_3_O_4_/rGO before and over 10,000 cycles at a 10 A g^−1^, Table S1: Comparison of the fabrication process and electrochemical performance of the as manganese oxide material in this work with those reported in previous literatures.
