# Supplementary material for: Rapid Production of Mn3O4/rGO as an Efficient Electrode Material for Supercapacitor by Flame Plasma
Source: Materials (Basel). 2018 May 24;11(6):881. doi: 10.3390/ma11060881 (PMC6025293; doi:10.3390/ma11060881)
Supplement: Supplementary file 1 [file materials-11-00881-s001.pdf]

materials

MDPI

Supplementary

Rapid Production of Mn_3O_4/rGO as an Efficient Electrode Material for Supercapacitor by Flame Plasma

Yang Zhou [1], Lei Guo [2,*], Wei Shi [2], Xuefeng Zou [1], Bin Xiang [1,3,*] and Shaohua Xing [4,*]

[1] School of Chemistry and Chemical Engineering, Chongqing University, Chongqing 400044, China; cquzhouyang@126.com (Y.Z.); njzouxf@126.com (X.Z.)
[2] School of Material and Chemical Engineering, Tongren University, Tongren 554300, China; truweishi@163.com (W.S.)
[3] National-municipal Joint Engineering Laboratory for Chemical Process Intensification and Reaction, Chongqing 400044, China
[4] State Key Laboratory for Marine Corrosion and Protection, Luoyang Ship Material Research Institute (LSMRI), Qingdao 26623, China
* Correspondence: cqglei@163.com (L.G.); xiangbin@cqu.edu.cn (B.X.); davy8006@126.com (S.X.)

Received: 7 May 2018; Accepted: 22 May 2018; Published: 24 May 2018

S1. Modified Hummers method

For oxidation step of synthesis, 23 mL of concentrated H_2SO_4 is placed into a 500 mL round-bottom flask and chilled to 0 °C using an ice bath. The natural flake graphite (1 g) and sodium nitrate (0.5 g) are added to the acid and stirred. 3g of $KMnO_4$ is added slowly and allowed to dissolve, with the temperature is closely monitored so as not to allow the mixture to go above 10 °C. This mixture is then allowed to react at 35 °C for 30 min, after which distilled water (50 ml) is added. The mixture is stirred for 15 min at 98 °C at which point an additional 140 mL of DI water is added. Shortly after the dilution with 140 mL of water, 10 ml of 30% H_2O_2 is added to the mixture resulting in a brilliant yellow color along with bubbling. The mixture is allowed to settle for at least 5 h after which the clear supernatant is decanted. The remaining mixture is centrifuged and washed with a total of 500 mL of 5% HCl solution followed by 1.5 L of DI water to remove the acid. The resulting solid is dried at 35 °C for 24 h.

S2. Preparation of activated graphene

The activated graphene was prepared via a modified microwave exfoliated method which has been reported by Rodney S. Ruoff previously.[1] Briefly, graphite oxide (GO) cake was synthesized from purified natural graphite (SP-1, Bay Carbon, Bay City, MI, USA) by the Hummers method. Microwave exfoliated graphite oxide (MEGO) was prepared by irradiating the GO in a microwave oven (GE) in ambient conditions. Upon microwave irradiation, the very large volumetric expansion of the GO cake yields a black and fluffy MEGO powder. The as-prepared MEGO powder was then dispersed and soaked in aqueous KOH solution, followed by filtration and drying, to form the MEGO/KOH mixture for chemical activation. The MEGO/KOH mixture was then put in a tube furnace under flowing argon at a pressure of about 400 Torr, and heated at 800 °C for one hour to enhancing their specific surface area.

S3. Mass measurement and calculation

The obtained MnO_2/GO film was filtered and dried under vacuum at 35 °C. Finally, the MnO_2/GO film were quickly passed through a naked flame from a burning alcohol lamp to obtain Mn_3O_4/rGO. After dried the Mn_3O_4/rGO ground into fine powder curing，the samples were directly used as an electrode, without adding any polymer binder or carbon black: the composite were

pressed between two nickel foams ($1 \times 1\ cm^2$) to form an electrode. The loading mass of the Mn_3O_4/rGO active materials can be obtained by measuring the weight change of the samples before and after the process of preparation electrode.

S4. The calculation of CV and GCD

Cyclic voltammetry (CV), galvanostatic charging/discharging (GCD), and electrochemical impedance spectroscopy (EIS) of the as-prepared samples were investigated on an electrochemical station (CHI760). The mass specific capacitance (C) were calculated from the CV and GCD curves according to the equations (1) and (2) below, respectively.

$$C = \frac{\int i(V)dV}{2mv\Delta U} \quad (1)$$

$$C = \frac{i\Delta t}{m\Delta U} \quad (2)$$

where *i* is the applied current, *v* is the scan rate, Δt is the discharge time, ΔU is the operating voltage window, *m* is the mass of the active material.

S5. The calculation of GCD of an ASC

The mass specific capacitance (C), mass specific energy density (E) and mass power density (P) were calculated from the GCD curves according to the equations below.

$$C = \frac{i\Delta t}{m\Delta U} \quad (3)$$

$$E = \frac{C(\Delta U)^2}{2 \times 3600} \quad (4)$$

$$P = \frac{E}{\Delta t} \quad (5)$$

where *i* is the applied current, Δt is the discharge time, ΔU is the operating voltage window, *m* is the total weight of the positive and negative electrodes active material.

S6.

Asymmetric supercapacitors were constructed using Mn_3O_4/rGO as a positive electrode, activated graphene (AG) as a negative electrode, and 0.1 M $NaSO_4$ as an electrolyte. To balance the charge storage, the masses of the positive and negative electrodes were optimized according to the following equation:

$$\frac{m_+}{m_-} = \frac{Cs_- \Delta V_-}{Cs_+ \Delta V_+} \quad (6)$$

According to the CV test at 20 mV s^{-1}, the optimized mass ratio between Mn_3O_4/rGO and AG should be m+/m− = 0.50449.

Supplementary Figures and Tables

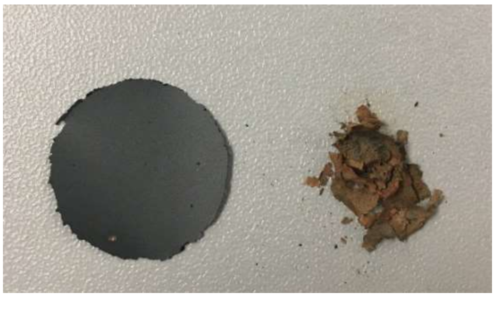

Figure. S1 The changes of the samples before and after the reaction triggered by the flame.

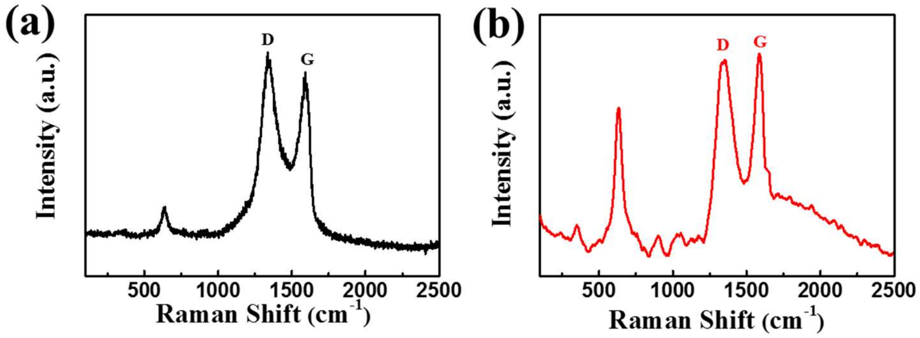

Figure. S2 Raman spectrums of MnO_2/GO and Mn_3O_4/rGO.

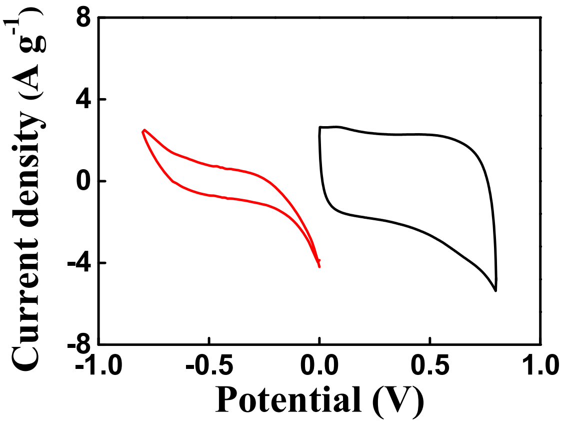

Figure. S3 CV curves of Mn_3O_4/rGO and AG at 20 mV s^{-1}.

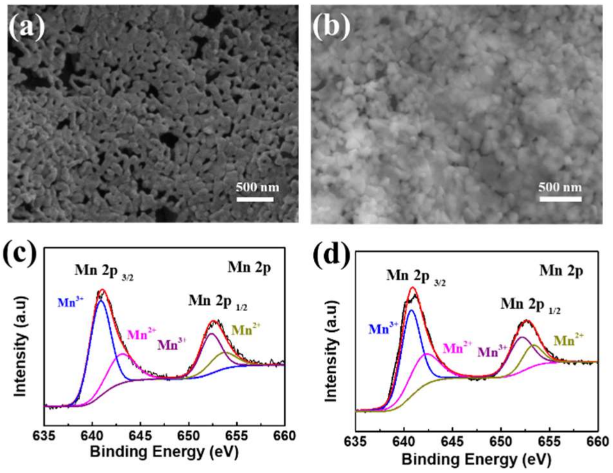

Figure. S4 SEM (**a**,**b**) and Mn 2p XPS spectra of Mn_3O_4/rGO composite (**c**,**d**) of Mn_3O_4/rGO before and over 10,000 cycles at a 10 A g^{-1}.

Table S1 Comparison of the fabrication process and electrochemical performance of the as manganese oxide material in this work with those reported in previous literatures.

Materials	Specific capacitance ($F\ g^{-1}$)	Current density/ scan rate	reference
MnO_2/C	257	0.5 A g^{-1}	[2]
MnOOH/rGO	327	0.1 A g^{-1}	[3]
MnO_x/rGO	164	2 A g^{-1}	[4]

Manganese oxide/carbon	191.1	0.1 A g^{-1}	[5]
Mn_3O_4@nitrogen graphene	158.9	10 mv s^{-1}	[6]
$MnCO_3$–rGO	335	2 A g^{-1}	[7]
MnO_2/rGO	122	0.2 A g^{-1}	[8]
Mn_3O_4/rGO	342.5	1 A g^{-1}	This work

References

1. Le, Q.J.; Wang, T.; Tran, D.N.H.; Dong, F.; Zhang, Y.X.; Losic, D. Morphology-controlled MnO2 modified silicon diatoms for high-performance asymmetric supercapacitors. *J. Mater. Chem. A* **2017**, *5*, 10856–10865.
2. Li, H.; Wang, B.; He, X.; Xiao, J.; Zhang, H.; Liu, Q.; Liu, J.; Wang, J.; Liu, L.; Wang, P. Composite of hierarchical interpenetrating 3D hollow carbon skeleton from lotus pollen and hexagonal MnO2 nanosheets for high-performance supercapacitors. *J. Mater. Chem. A* **2015**, *3*, 9754–9762.
3. Sun, S.; Wang, S.; Xia, T.; Li, X.; Jin, Q.; Wu, Q.; Wang, L.; Wei, Z.; Wang, P. Hydrothermal synthesis of a MnOOH/three-dimensional reduced graphene oxide composite and its electrochemical properties for supercapacitors. *J. Mater. Chem. A* **2015**, *3*, 20944–20951.
4. Wang, Y.; Lai, W.; Wang, N.; Jiang, Z.; Wang, X.; Zou, P.; Lin, Z.; Fan, H.; Kang, F.; Wong, C.; Yang, C. A reduced graphene oxide/mixed-valence manganese oxide composite electrode for tailorable and surface mountable supercapacitors with high capacitance and super-long life. *Energy Env. Sci.* **2017**, *10*, 941–949.
5. Wang, R.; Ma, Y.; Wang, H.; Key, J.; Brett, D.; Ji, S.; Yin, S.; Shen, P.K. A cost effective, highly porous, manganese oxide/carbon supercapacitor material with high rate capability. *J. Mater. Chem. A* **2016**, *4*, 5390–5394.
6. Liu, L.; Su, L.; Lang, J.; Hu, B.; Xu, S.; Yan, X. Controllable synthesis of Mn_3O_4 nanodots@nitrogen-doped graphene and its application for high energy density supercapacitors. *J. Mater. Chem. A* **2017**, *5*, 5523–5531.
7. Jana, M.; Samanta, P.; Chandra Murmu, N.; Kuila, T. Morphology controlled synthesis of MnCO3–RGO materials and their supercapacitor applications. *J. Mater. Chem. A* **2017**, *5*, 12863–12872.
8. Miniach, E.; Sliwak, A.; Moyseowicz, A.; Fernández-Garcia, L.; González, Z.; Granda, M.; Menendez, R.; Gryglewicz, G. MnO2/thermally reduced graphene oxide composites for high-voltage asymmetric supercapacitors. *Electrochim. Acta* **2017**, *240*, 53–62.

© 2018 by the authors. Submitted for possible open access publication under the terms and conditions of the Creative Commons Attribution (CC BY) license (http://creativecommons.org/licenses/by/4.0/).
